# Editorial: Redox regulation and signaling in neurodegenerative diseases

**DOI:** 10.3389/fnagi.2023.1135303

**Published:** 2023-01-17

**Authors:** M. I. Holubiec, M. Gellert, E. M. Hanschmann

**Affiliations:** ^1^IBioBA-MPSP Instituto de Investigación en Biomedicina de Buenos Aires, Partner Institute of the Max Planck Society, Buenos Aires, Argentina; ^2^Institute for Medical Biochemistry and Molecular Biology, University Medicine Greifswald, University Greifswald, Greifswald, Germany; ^3^Independent Researcher, Essen, Germany

**Keywords:** redox regulation, neurodegenerative diseases, Alzheimer's disease, Parkinson's disease, ferroptosis, mitochondria, metabolism

The production of reactive oxygen, nitrogen, and sulfur species plays a key role in intra- and intercellular signal transduction (Ray et al., 2012; Angelova and Abramov, 2018; Nissanka and Moraes, 2018). As second messengers, these molecules are enzymatically produced and they are essential for regulation of posttranslational modifications of proteins, biochemical pathways, and cellular functions (Dröge, 2002). The identification and characterization of redox molecules, redox-regulated pathways, and functions in different brain areas has also shed light on the origin of oxidative distress and neurodegeneration in the central nervous system (Patten et al., 2010). In fact, there is increasing evidence suggesting that redox changes occur in the early stages of neurodegenerative diseases such as Amyotrophic lateral sclerosis, Alzheimer's (AD), Huntington's, and Parkinson's disease (PD). However, different brain areas are affected and the biochemical and pathophysiological characteristics they display are distinct from one another (Patten et al., 2010; Angelova and Abramov, 2018). To this day, the causes that give rise to the hallmarks and symptoms of many neurodegenerative pathologies remain mostly elusive, however, oxidative distress has been linked to neurodegeneration and -inflammation. We believe that it is important to better understand redox biology on a molecular, biochemical, and cellular level and also, to translate this knowledge to the clinics.

The purpose of this collection of articles is to better comprehend the nature of neurodegenerative pathologies in the light of redox biology. Our Research Topic features two original articles on patients suffering from PD and AD, respectively. Both studies aim to identify new extracellular mediators that correlate with symptoms present in neurodegenerative diseases. The study of extracellular redox-modified biomolecules in biomedicine can be of outmost importance to find clear connections between molecular mechanisms and pathophysiological outcomes (Loreto Palacio et al., 2022). Li et al. analyzed different reactive species, superoxide dismutase (SOD) and tumor necrosis factor- alpha (TNF-alpha) in cerebrospinal fluid (CSF) from patients suffering from PD. The levels of hydrogen peroxide (H_2_O_2_) and nitric oxide (NO) were elevated and positively correlated with the presence of neuropsychiatric symptoms, whereas the levels of total SOD and TNF-alpha were decreased and negatively correlated with these symptoms. Interestingly, Tau levels in the CSF of these patients positively correlated with H_2_O_2_, NO, and neuropsychiatric symptoms. A negative correlation was found for Tau and TNF-alpha. The authors propose that the elevated levels of H_2_O_2_ and NO indicate oxidative distress and are linked to neurodegeneration. Antioxidant therapies could be a therapeutic approach when treating PD patients (Ciulla et al., 2019; Church, 2021). Amick et al. determined bioenergetic differences and plasma circulating factors in patients with normal cognition (NC), mild cognitive impairment (MCI), and dementia due to probable AD (DEM). Mitochondrial respiration in peripheral blood mononuclear cells (PBMCs) was lower in patients with DEM. Interestingly, treatment of naïve Neuro-2a cells with patient serum modulated the bioenergetics of the cells according to the bioenergetic capacity of donor PBMCs. The authors identified two circulating lipids by mass spectrometry, glycocholic acid and linoleic acid, that were significantly altered in DEM patients and correlated with the PBMC and Neuro-2a data. They conclude that circulating factors present a clear effect on mitochondrial bioenergetics and that levels of these factors are different in patients with dementia compared to patients without.

Three reviews are focused on different but interconnected processes that are altered in neurodegenerative diseases, including signal transduction, metabolism, mitochondrial (dys)function and ferroptosis. We (Holubiec et al.) reviewed the most important advances in redox signaling and metabolism in AD, highlighting disease onset and progression. Changes in redox signaling in AD are related to the increase of different reactive oxygen species such as H_2_O_2_, ${\text{O}}_{2}^{•-}$, decreased levels or activities of antioxidant enzymes, abnormal oxidation of macromolecules linked to elevated amyloid beta (Aβ) production, and changes in mitochondrial homeostasis and Tau phosphorylation. We particularly emphasize the specific modifications of cysteinyl residues in key proteins related to metabolic pathways that are altered in AD. A small but comprehensive summary of the experimental models used in AD research and redox biology complements the review article.

Canal et al. propose a close relation between neurodegenerative diseases and impaired fasting glucose (IFG). The latter is characterized by an increase of blood glucose levels due to an inability to utilize insulin. IFG does not have one particular cause, however, there are many contributing factors such as metabolic syndrome, obesity, smoking, and sedentarism (Swiecicka-Klama et al., 2021). Different studies have shown a correlation between type 2 diabetes mellitus (T2DM) and different neurodegenerative diseases. The authors suggest a link between metabolic changes in T2DM and cognitive decline, presenting a relationship between neurodegeneration, high glucose levels and advanced glycation end products, mitochondrial alterations, decrease of antioxidant enzymes, such as SOD2, and inflammation. The relation between metabolism and different neurodegenerative diseases has been addressed from different angles in the past (Ludolph et al., 1993; Quansah et al., 2018; Tu et al., 2019). This review focuses on the early changes occurring in T2DM that could lead to neurodegeneration, which makes it novel and particularly interesting.

Finally, Sun et al. wrote an interesting review on ferroptosis in neurodegenerative diseases, which are generally characterized by chronic progressive neuronal degeneration and synaptic loss (Dugger and Dickson, 2017). Their etiology is complex and diverse and a common link is mitochondrial dysfunction (Lin and Beal, 2006). Moreover, in recent years ferroptosis has been associated with these pathologies (Reichert et al., 2020; Song and Long, 2020), providing new explanations and mechanisms for their occurrence and progression. Sun et al. provide a thorough overview of ferroptosis related mechanisms and its relation to neurodegenerative pathologies. It is the authors belief, and ours, that further understanding of these mechanisms can shed light on the etiology of neurodegenerative diseases leading to the development of novel therapeutic strategies that are so urgently needed.

## Author contributions

MH compilated and made the first summary of the contributions to the current special issue. EH and MH wrote the main body of the editorial. MG wrote and edited the bibliography. EH, MH, and MG corrected and edited the editorial and made the final changes and remarks to the whole manuscript. All authors contributed to the article and approved the submitted version.
